# Prey Switching and Natural Pest Control Potential of Carabid Communities over the Winter Wheat Cropping Season

**DOI:** 10.3390/insects15080610

**Published:** 2024-08-13

**Authors:** Ambre Sacco--Martret de Préville, Karin Staudacher, Michael Traugott, David A. Bohan, Manuel Plantegenest, Elsa Canard

**Affiliations:** 1 IGEPP, INRAE, Institut Agro, Université de Rennes, 35653 Le Rheu, France; elsa.canard@inrae.fr; 2 Functional Diversity in Agro-Ecosystems, Crop Research Institute, Drnovská 507, Ruzyně, 161 06 Praha 6, Czech Republic; 3 Mountain Agriculture Research Unit, Institute of Ecology, University of Innsbruck, Technikerstraße 25, 6020 Innsbruck, Austria; karin.wastian@sinsoma.com (K.S.); michael.traugott@uibk.ac.at (M.T.); 4 Agroécologie, AgroSup Dijon, INRAE, Université de Bourgogne Franche-Comté, 17 rue Sully, BP 86510, 21065 Dijon CEDEX, France; david.bohan@inrae.fr; 5 IGEPP, INRAE, Institut Agro, Université de Rennes, 35000 Rennes, France

**Keywords:** food web, pest control, intraguild predation, alternative prey, molecular gut content analysis, multiplex PCR diagnostic

## Abstract

**Simple Summary:**

Carabids are abundant and diverse arthropods in agroecosystems and are well known for their generalist diet and therefore biocontrol potential. Most studies on carabids’ diet have focused on periods of peak pest infestations but their feeding habits through the cropping season are poorly known. In this study, we explored how carabids’ trophic interactions with pests (aphids and slugs) and alternative prey (spiders, earthworms and springtails) varied from the wheat seedling emergence to wheat ripening by analysing the prey DNA in their gut. Carabids seemed to switch between prey depending on their availability at the time, reaffirming carabids’ opportunistic diet. Regarding biocontrol, carabid taxa contributed complementarily to pest predation over the season and per pest taxon, highlighting the importance of favouring diversity in agroecosystems.

**Abstract:**

To date, evaluating the diets of natural enemies like carabids has largely been limited to spatially explicit and short-term sampling. This leaves a knowledge gap for the intra-annual dynamics of carabid diets, and the provision and timing of delivery of natural pest control services. Season-long pitfall trapping of adult carabids was conducted in conventional winter wheat fields, from November 2018 to June 2019, in five French departments. Diagnostic Multiplex PCR of carabid gut contents was used to determine the dynamics of carabid diets. The overall detection rate of target prey DNA was high across carabid individuals (80%) but varied with the prey group. The rate of detection was low for pests, at 8.1% for slugs and 9.6% for aphids. Detection of intraguild predation and predation on decomposers was higher, at 23.8% for spiders, 37.9% for earthworms and 64.6% for springtails. Prey switching was high at the carabid community level, with pest consumption and intraguild predation increasing through the cropping season as the availability of these prey increased in the environment, while the detection of decomposer DNA decreased. Variation in diet through the cropping season was characterized by: (i) complementary predation on slug and aphid pests; and (ii) temporal complementarity in the predominant carabid taxa feeding on each pest. We hypothesize that natural pest control services delivered by carabids are determined by complementary contributions to predation by the different carabid taxa over the season.

## 1. Introduction

Biological control theory posits that the efficiency of pest control depends on natural enemy abundance and diversity [1], as well as the complementarity of their contribution to control, including attacking different species and life stages of pests and facilitating prey capture by other natural enemies [2]. For instance, population growth of the weed *Alopecurus myosuroides* can be controlled by complementary seed predation by vertebrates and invertebrates in Swedish winter wheat fields [3]. The taxa contributing most to pest control may vary throughout the cropping season [2]. In addition, the presence of alternative prey to pests in their absence can be critical to maintaining large populations of natural enemies [4]. Therefore, the effectiveness of biocontrol services may depend on both the turnover of predator taxa and the presence of alternative prey in agricultural fields. Deciphering the temporal variation in trophic interactions between predators and their prey, both pests and non-pests, is essential to assess the switch in their diet over the agricultural season and evaluate the dynamics of pest control services in agroecosystems.

Carabids are among the most abundant and diverse families of arthropod generalist predators in temperate agricultural fields. It has been proposed that the polyphagous and opportunistic diet of carabids may make them good natural regulators of a wide range of pests [5,6]. Indeed, carabids have been observed preying on weed seeds [7] and a wide variety of invertebrate pests [8,9]. Carabids also consume alternative resources that could be beneficial organisms, including decomposers [7,10] and other natural enemies, causing intraguild predation [11,12]. Beyond these disservices caused by the direct predation on beneficial organisms, alternative prey might also divert carabids from pest control [13]. Conversely, alternative prey can ensure the presence and aggregation of carabids in agroecosystems by acting as alternative food resources and sustaining them during periods of low pest prey availability [13]. Our expectation is that the diversity of carabid species, when combined with the breadth, complexity and variation of their diet, makes carabids a model community of predators for studying the balance of ecosystem services and disservices provided by generalist natural enemies. In addition, it may highlight the indirect role played by non-pest prey in supporting biocontrol services as alternative food sources for generalist predators.

Carabid diet and feeding preferences have been studied in the laboratory using panels of prey in cafeteria or voracity tests [10,14]. These laboratory tests can provide repeatable information on predatory behaviour by simulating competition or risk of intraguild predation [15] but they do not mirror the frequency of prey–predator interactions under natural conditions [16]. In the field, diets have been inferred by observing the population dynamics of carabid prey [17,18] or indirectly by using predator exclusion cages [19]. Such methods provide only correlative data, however, and are frequently unable to pinpoint specific prey–predator interactions for which the test is evidence of feeding [20]. Molecular gut content analyses (MGCAs), and in particular diagnostic multiplex PCR, offer a direct test for unravelling specific predator–prey trophic interactions [12,20].

Diagnostic multiplex PCR has been successfully used to determine the diet of the most abundant carabid species and made it possible to sample for short-term, spatially explicit variation in carabid diets. This has included examining whether trophic interactions change with farming system [9,21], habitat heterogeneity [22] and pest abundance [12]. This sampling has focussed only on specific periods of the cropping season, such as periods of pest presence (but see [but [11]]), and therefore overlooked the important role of alternative prey in the carabid diet, particularly when pests are absent from fields. Indeed, as alternative food resources, alternative prey can provide a complementary indirect ecosystem service in maintaining natural enemy populations [23], which can only be evaluated by following diets through time. Furthermore, focusing on specific periods of the cropping season excludes sets of carabid species from consideration.

Our approach is to work at the community level, explicitly highlighting the dynamics of the trophic ecology of the entire carabid community and their predation on pests and alternative prey. Our aim is to determine which carabid species are the most effective predators of pests, when this predation take place and what other resources are involved in the population cycle.

In the present study, we determine carabid species’ abundance and their diets over the entire cropping season of conventional winter wheat crops in France. Using multiplex PCR, we designed an approach to evaluate trophic interactions between carabids and five groups of potential prey, including problematic pests of winter wheat and alternative prey, natural enemies (intraguild predation) and decomposers.

The multiplex PCR is applied to the entire carabid community sampled to estimate the diet of carabids and thereby the services provided over the wheat cropping season. Following the expectation of Gray et al. [24], we assume that prey consumption will be primarily determined by the presence and abundance (availability) of prey resources in the field. Hence, we hypothesise that prey consumption increases with prey availability (i). As pest and spider abundance increases over the wheat cropping season [11,12], we expect their consumption by carabids will increase accordingly (ii). We also expect the predation rates on decomposers to remain more stable throughout the season, given their near-constant availability (iii). These contrasting dynamics lead us to predict switching in the prey eaten by the carabids over the cropping season, and especially around the time of peak pest availability.

## 2. Materials and Methods

### 2.1. Field Sampling

Across five regions of France, carabids were collected in ten conventional wheat fields, with two fields per region (Supplementary Material S1): Essonne (Île-de-France), Ille-et-Vilaine (Bretagne), Loire-Atlantique (Pays de la Loire), Vendée (Pays de la Loire) and Meurthe-et-Moselle (Lorraine). The regions of study lay on a climatic, landscape and farmland gradient. Ille-et-Vilaine, Loire-Atlantique and Vendée are oceanic regions with a dense hedgerow network across the farmland (“bocage” structure) dominated by a polyculture-livestock system. The Essonne and Meurthe-et-Moselle regions have an altered oceanic and semi-continental climate, respectively, and their farmland mainly exhibits cereals in open fields. The two selected fields per region were chosen randomly across the farmland. Field size ranged from 0.5 ha for the smallest field to 9.5 ha for the largest one.

Each field was sampled in each of five sampling sessions (November 2018, December 2018, April 2019, May 2019 and June 2019), using pitfall traps (9.5 cm diameter, 520 mL volume). Sampling sessions were designed depending on the growth stage of the wheat and periods of activity of invertebrates. Autumn sessions (November and December 2018) are periods of emergence and post-emergence of wheat seedlings when plants are sensitive to slugs. The session in early spring (April 2019) corresponds to arthropod activity resuming after the winter. The May session is linked to the main colonization period of cereal aphids. The last session in June is associated with wheat ripening, a period when aphid infestation reaches its peak. In each field, 25 dry pitfall traps were opened and emptied twice a day (morning and late afternoon) for three consecutive days. No trapping was carried out in the Meurthe-et-Moselle region in the November and December 2018 sampling sessions.

The pitfall traps were filled with clay pellets to provide cryptic refuges that can reduce predation among the captured animals. At emptying, all trapped carabids were placed on ice before being transferred to individual 2 mL Eppendorf tubes upon arrival in the laboratory. Carabids were stored at −20 °C as fast as possible to stop the digestion process, but a first rapid identification occurred on the live carabids to the genus level or morphotypes to have an idea of the community structure.

### 2.2. Carabids Selection for MGCA

The November and December 2018 sessions were pooled for analysis due to the low abundance of carabids trapped during this cold period. These pooled samples are henceforth referred to as the autumn session.

At each trapping point (i.e., a trapping session in a specific field), 48 individuals from among all the trapped carabids were sub-sampled for MGCA. Each species was sub-sampled according to its proportion in the entire community so that they constituted a representative subsample of the local trapped communities. Where fewer than 48 individuals were trapped (13 trapping points out of 38), all the individuals were retained for molecular analyses. Where possible, all beetles selected for MGCA were re-identified with Roger et al.’s identification key [25] to the species level, but were otherwise identified to genus, while making sure they did not thaw out. All subsampled beetles were then placed individually in individual 1.5 mL Eppendorf tubes on ice.

The wheat-field specialist, *Poecilus cupreus*, was overwhelmingly dominant at many sampling points. To ensure a specific diversity in the sample, a maximum of 30 individuals belonging to this species was included for each point. Where necessary, a weighting was used to account for this deviation from the pro rata sampling.

### 2.3. DNA Extraction

To reduce the risk of contamination, all extractions were carried out in a sterilised, pre-PCR laboratory. For those taxa smaller than 15 mm, the whole body of each carabid was processed for DNA extraction. For taxa longer than 15 mm, such as *Pterostichus melanarius* and *Carabus* spp., the gut was dissected and processed for DNA extraction.

To extract DNA, each carabid was transferred into an individual 2 mL Eppendorf tube filled with lysis buffer, 10 µL of Proteinase K (20 mg/mL) and a volume of TES adjusted to carabid size (0.1 M TRIS, 10 mM EDTA, 2% SDS, pH 8). TES volume was 290 µL for carabid species smaller than 4 mm, 630 µL for *P. melanarius*, 990 µL for *Carabus* spp. and 430 µL for all other species. Cuticles and soft tissues were crushed and homogenised with glass beads of various sizes (two cycles of 1 min at 5000 RPM in a Precellys^®^ 24 Tissue Homogenizer, Bertin Technologies, Montigny-le-Bretonneux, France). The samples were centrifuged at 800 RPM for 1 min and incubated overnight at 58 °C. DNA was extracted using the BioSprint 96 DNA Blood Kit (Qiagen, Hilden, Germany) in a BioSprint 96 device (Qiagen) in accordance with manufacturer’s instructions. An amount of 200 µL of DNA extract was obtained per sample and stored at −20 °C until PCR amplification.

Four negative controls per batch of 96 samples were included to check for DNA contamination (two at the lysis step and two at the extraction step).

### 2.4. Primer Design, Multiplex PCR Assay and PCR Amplification

A multiplex PCR assay was designed based on previously published group-specific primer pairs for earthworms, springtails, aphids and spiders, and a newly designed pair for slugs (Supplementary Material S2). Primer Premier 5 (PREMIER Biosoft International, Palo Alto, CA, USA) was used to design primers targeting slug 18S DNA. Primer sensitivity and specificity evaluation was performed for all primers pairs during the multiplex assay (Supplementary Material S3).

The new multiplex assay was conducted on a 10 µL volume containing 5 µL of QIAGEN Multiplex PCR Mastermix (Qiagen), 1 µL of 10× Primer mix (for final primers concentration in PCR see Supplementary Material S2), 1.5 µL of DNA extract, 0.5 µL of BSA (10 mg/mL, Qiagen) and 2 µL of PCR water to adjust the volume.

PCR cycling conditions included three steps: an initial cycle of 15 min at 95 °C, then 35 cycles of 30 s at 94 °C, 90 s at 63.5 °C and 60 s at 72 °Cand finally a 10 min-cycle at 72 °C. PCR products were displayed using the QIAxcel electrophoresis system (Qiagen) following the protocol of Staudacher et al. [12]. 

### 2.5. Statistical Analysis

All statistical analyses were carried out with the software R [26] version 3.6.1.

The DNA detection rate of a specific prey was calculated as the number of carabid individuals for which this prey DNA was detected in their gut divided by the total number of individuals that were positive for at least one of the five tested prey. The DNA detection rate was then modelled as a mixed generalized linear model with prey group (five levels: aphid, earthworm, slug, spider, springtail), session (four levels: autumn, April, May, June) and their interaction as fixed effects and with the field, nested in the region, and individual carabid codes as random effects. The model was fitted with the “glmer()” function from the “lme4” package (v. 1.1.35.1) [27], using a binomial distribution and the logit link function. Detection of overdispersion was tested using Pearson residual χ^2^ statistic with the “simulateResiduals()” function from the “DHARMa” package (v. 0.4.6) [28]. Significance of the explanatory variables was checked with the “Anova()” function from the “car” package (v. 3.1.2) [29]. Pairwise comparisons between the levels of each factor were carried out using “emmeans()” and “pairs()” functions from the “emmeans” package (v. 1.10.0)to check the significance of detection rate differences between sessions [30].

As we limited the number of *P. cupreus* individuals retained for molecular analysis to a maximum of 30 at each sampling point, we created a corrected version of the dataset respecting the observed relative abundance of this species in the pitfall traps. This was achieved by randomly resampling the appropriate number of *P. cupreus* among the 30 tested individuals. For the sake of clarity, this dataset is henceforth termed the “community dataset” and it is used uniquely for analyses carried out at the community level.

The “plotweb()” function from the “bipartite” package (v. 2.19) was used to graph trophic interaction networks (food webs) of carabid communities and their dynamics over the wheat cropping season [31].

At the carabid genus level, patterns of temporal diet variation were drawn using individuals positive for at least one prey group. Seasonal predation profiles, defined as the list of predation rates for each combination of prey and session, were assessed for the most abundant genera only (>40 individuals). Consequently, only nine carabid genera were considered: *Amara*, *Anchomenus*, *Bembidion*, *Brachinus*, *Harpalus*, *Nebria*, *Notiophilus*, *Poecilus* and *Trechus*. Similarities and specificities between seasonal predation profiles of those genera were studied by Principal Component Analysis using the “PCA()” and “explor()” functions of the “FactoMineR” (v. 2.10) [32] and “explor” (v. 0.3.10) [33] packages. The procedure was followed by a hierarchical clustering using the “HCPC()” function to group selected genera in clusters of similar predation profiles and visualized with the “fviz_dend()” function, both from the “FactoMineR” package (v. 2.10) [32]. From the list of outputs of the “HCPC()” function, “$desc.var” provided the levels of factors that significantly contributed to describing each cluster.

## 3. Results

### 3.1. Carabid Communities

A total of 5843 carabids were trapped during sampling among which 1819 were processed for MGCA. A total of 57 taxa were identified, with abundances ranging between one and 625 individuals (details in Supplementary Material S4).

### 3.2. Detection Rates at Community Level

A total of 80% of processed carabid individuals tested positive for at least one prey in the multiplex PCR. Across all carabids examined, the detection rates for pests were 8.1% for slugs and 9.6% for aphids. Spider DNA was detected in 23.8% of carabids. Of all carabids, 37.9% contained decomposer DNA, 37.9% of earthworm and 64.6% of springtail. Detection rates for prey varied depending on region: from 7.1% in Meurthe-et-Moselle to 12.5% in Vendée for aphids, from 0.9% in Meurthe-et-Moselle to 10.4% in Ille-et-Vilaine for slugs, from 11.8% in Essonne to 45.5% in Vendée for spider, from 52% in Essonne to 72% in Loire-Atlantique for springtails and from 20.0% in Essonne to 48.0% in Meurthe-et-Moselle for earthworms.

**Figure 1:**
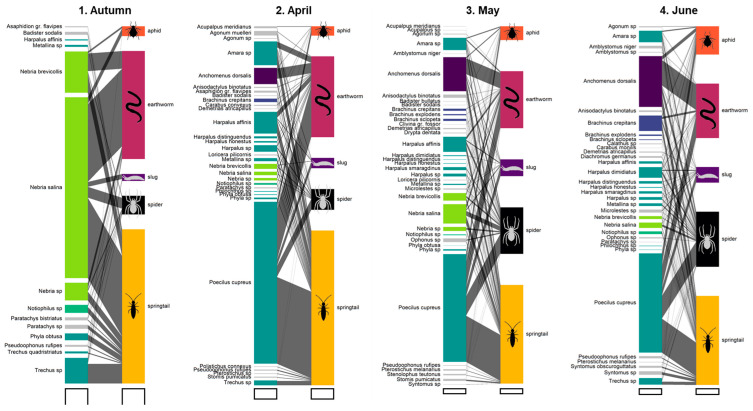
Bipartite food webs showing the number of individuals of each carabid taxon testing positive for DNA of each prey type over the wheat cropping season. The width of the left-hand blocks represents the number of DNA detections for each taxon of carabids. The width of the right-hand blocks represents the number of DNA detections for each prey group. The width of the link represents the number of DNA detections for each prey for each carabid taxon. The colours of the carabid taxa and prey groups are consistent from one figure to the next. White rectangles outlined in black under the food webs all represent 20 DNA detections.

The number of carabids which tested positive to pest and spider DNA increased through the season, while the number of carabids positive to decomposer DNA peaked in spring (April–May) (Figure 1).

### 3.3. Factors Influencing Prey DNA Detection Rates at Carabid Community Scale

Prey group, session and their interactions all have a significant effect on the probability of prey DNA detection in carabids (Table 1).

Overall, detection rates increased over sessions from autumn to summer, but not for all prey types. Significant increases in detection of pest DNA were observed (aphid: autumn < June, with *p* < 0.001, April < (May, June), with *p* < 0.01 and *p* < 0.001, respectively, and May < June, with *p* < 0.001; slug: autumn < (May, June), with *p* < 0.01 and *p* < 0.05, respectively, and April < (May, June), with *p* < 0.001 and *p* < 0.05, respectively) and of spider DNA (spider: (autumn, April) < (May, June) with *p* < 0.001 in all cases). Detection of earthworm DNA significantly decreased from autumn to summer (earthworm: autumn > (April, June) with *p* < 0.05 and *p* < 0.001, respectively). Detection of springtail DNA remained generally high, except for a significant drop in June (springtail: (autumn, April, May) > June, with *p* < 0.001 in all cases).

**Figure 2:**
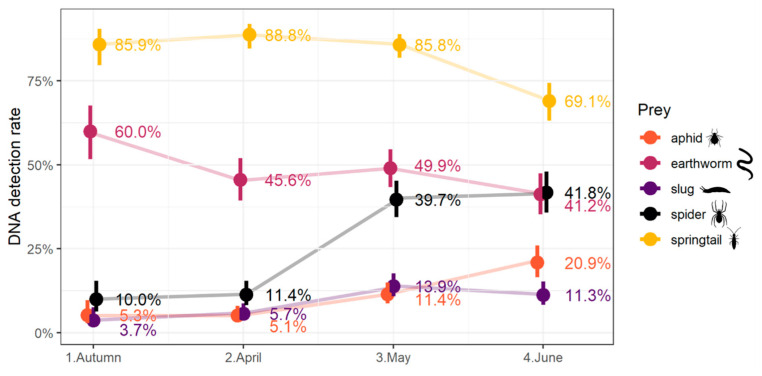
Variations throughout the cropping season of the DNA detection rate in carabid beetle communities for each prey group.

The overall detection rates and their seasonal changes are represented in Figure 2. Results from the “community dataset”, obtained by resampling, were broadly similar.

### 3.4. Seasonal Predation Profile of Predominant Carabid Genera

**Figure 3:**
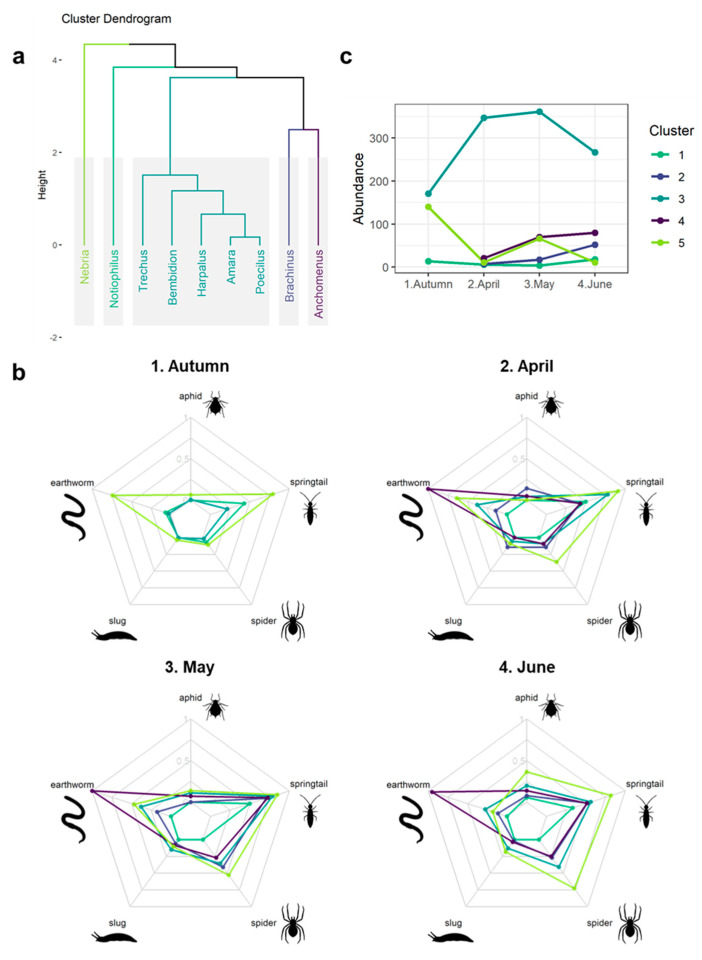
Cluster analyses of the most abundant carabid genera based on their predation profile, i.e., their seasonal predation patterns, (**a**), DNA detection rates for each combination of prey and cluster by session (**b**) and abundance variations of carabid genera grouped by clusters throughout the cropping season (**c**). The dendrogram presents the clustering of carabid genera based on the similarities in the seasonal pattern of prey DNA detection rates. Colours indicate clusters (green: cluster 1, indigo: cluster 2, blue: cluster 3, violet: cluster 4, light green: cluster 5). The absence of a specific colour on diamond plot (**b**) indicates that no individuals of the cluster associated with the colour were tested.

Using hierarchical clustering, the nine most frequent genera were grouped into five clusters based on the seasonal variation pattern of their diet (Figure 3). Cluster 1 was solely composed of the *Notiophilus* genus and its diet did not differ significantly from the average at any period. Significantly higher rates of DNA detection of the two pests in April characterised cluster 2 (aphid: *p* < 0.01 and slug: *p* < 0.05), which was also formed of a single genus, *Brachinus*. Cluster 3 was the only multi-genera cluster (*Amara*, *Bembidion*, *Harpalus*, *Poecilus*, *Trechus*) and was characterised by a significantly higher rate of slug DNA detection in May (*p* < 0.05). Cluster 4 included only the *Anchomenus* genus and was distinguished by significantly higher rates of earthworm DNA detection in April (*p* < 0.05), May (*p* < 0.05) and June (*p* < 0.05), and a lower springtail consumption rate in April (*p* < 0.05). Finally, the *Nebria* genus composed cluster 5 and had significantly higher rates of earthworm and aphid DNA detection in autumn (*p* < 0.01, *p* < 0.05), spider in April (*p* < 0.05) and aphid in June (*p* < 0.05).

## 4. Discussion

The present study suggests a prey switching in carabids’ diet consistent with prey availability in the environment. This relationship highlights the importance of alternative prey in sustaining carabid populations in the absence of pests. Temporal and complementary predation were also unveiled among carabid taxa through their dietary specificities.

This study corroborates previous findings that carabids prey upon two major wheat pests, aphids and slugs. However, detection rates of pest DNA were low compared to those of alternative prey DNA. We expected that pest detection rates would match the period of pest availability and be highest during peak abundance [12,21]. Indeed, aphid DNA detection increased sharply over the cropping season, to a peak in June, in a similar way to aphid infestation in the field (see Supplementary Material S5 for details), as has been found in previous studies [12,21]. The slug period of activity extends from November to June, with a peak in spring [9,34], and as expected slug DNA detection also peaked in April–May. However, this pest is a major crop pest in autumn, when they can attack wheat seedlings, while slug DNA detection remained low in autumn. This pattern might be due to a low slug infestation in 2018 (see Supplementary Material S5 for details) but also to the composition of carabid assemblages during the cold season. *Nebria* was the most abundant carabid genus during this period, but had low rates of slug consumption, in agreement with a previous result in the literature [9].

Despite their predation on a wide variety of pests, the generalist diet could result in an overall decrease in pest consumption due to intraguild predation. Indeed, in May and June, 40% of tested carabids were positive to spider DNA. As expected, this increase is positively correlated with spider availability in the environment (see Supplementary Material S5 for details). Intraguild predation rate on spiders was higher in this study than in most precedent works [12,21], but was consistent with the results of Davey et al. [11]. Interestingly, Davey et al. [11] showed that such high predation rates did not reduce the spider population, which continued to increase, potentially mitigating the risk of disservice resulting from intraguild predation by carabids. This is consistent with previous meta-analyses that have suggested that antagonistic interactions between natural enemies do not always interfere with biocontrol services [35,36].

Carabids were found consuming other service providers such as decomposers: DNA detection rates as high as 37.9% for earthworms and 64.6% for springtails were obtained during this analysis, which is at least double what was observed in previous studies [7,12,21]. Such differences might be due to the low infestation rates of pests in French fields in 2018–2019 resulting in carabids using decomposers as alternative prey [23]. Springtail DNA detection rates decreased slightly at the end of cropping season but remained high for most carabid species. Springtails are considered the basal prey of several carabid species [37], and our study corroborates these expectations. Decomposers are present and abundant all year round in French fields and, as alternative prey, might support carabid populations in seasons when pest availability is low. Settle [38] found that detritus-feeding and plankton-feeding insects were alternative prey for generalist predators and sustained their populations in rice fields early in the growing season. Yoo [4] observed that thrips were alternative prey of the generalist predator stinkbug, *Orius insidiosus*, facilitating its earlier colonization of fields. In both cases, the presence of alternative prey was believed to provide sufficient food resources to natural enemies to give them a head start in suppressing subsequent pest outbreaks [4,38]. In our study, earthworms and springtails are the predominant components of the carabid diet, but their detection rate declines over the cropping season in favour of pests and spiders. We argue, therefore, that alternative prey might contribute indirectly to pest consumption by sustaining carabid populations when pest prey is scarce. Carabids of the genus *Anchomenus* appeared as something of an outlier to this pattern. The detection rate of earthworm DNA in *Anchomenus* remained consistently above 95% throughout the year, suggesting a strong preference for earthworms. This preference has not previously been demonstrated. Kamenova [39] has shown that the carbon isotopic signature of *Anchomenus dorsalis* is linked to the isotopic signature of the crop, suggesting feeding on crop herbivores. This apparent difference may be an effect of prey communities differing between studies, but it is still consistent with our results because while high rates of earthworm DNA were detected in *Anchomenus* in our study, presumably due to their elevated availability, our results indicate that *Anchomenus* also eats all other prey tested and is not a strict earthworm specialist.

In addition, our results highlight the complementarity contribution of different carabid species to pest predation throughout the cropping season and the importance of considering their diversity to properly assess their potential. Indeed, for the two pest groups we examined, the carabid species providing the most predation changed over time. *Nebria* and *Brachinus* contributed to predation in a relay, while the taxa providing the greatest predation of slugs changed from *Brachinus* to a set of other genera (*Amara*, *Bembidion*, *Harpalus*, *Poecilus* and *Trechus*). *Nebria* outperformed other carabid genera in both the autumn and early summer, suggesting that this genus has the potential to predate a lot of aphids in wheat crops at two different times of the season, possibly autumn aphids and grain aphids, respectively. This is consistent with previous carbon isotopic analysis which suggested that *Nebria salina* consumed prey feeding on crop plants [39]. *Nebria* did not prey upon slugs in the autumn. Adult slugs protect themselves from predator attacks by secreting mucus, and *Nebria brevicollis* has been found to prey less on live slugs than other species of carabids, such as *Pterostichus madidus*, and with a success rate that decreases with slug size [40,41] but see also [42]. In the light of those results, we find no evidence from our fields that carabids importantly consume slugs in autumn, when they can cause damage in winter-sown crops. However, the increase in slug DNA detected in carabids in the spring, including for *Nebria*, suggests that carabids do consume slugs, but maybe at specific life stages. Indeed, other species of carabids, *Poecilus cupreus* and *Pterostichus melanarius*, have been observed consuming eggs of *Deroceras reticulatum* and *Arion lusitanicus* in laboratory conditions [41], suggesting that some control may be exerted by carabids on early life stages of slugs. Interestingly, in spring, genera of smaller carabids, including *Bembidion* and *Trechus*, had similar slug consumption rates to the larger-bodied genera, suggesting that they also contribute to slug predation. In addition to temporal complementarity of predation by the carabid community, our data suggest complementarities of pest predation. In spring, *Brachinus* showed evidence of complementary predation for both aphids and slugs. *Amara* and *Harpalus* have generally been considered to be granivores and to date studies have focused on their ability to control weeds by consuming their seeds [43]. Our results indicate that they also contribute to predation of slugs. However, the parasitic behaviour of *Brachinus* on the carabid genus *Amara* [44] could potentially interfere with its pest and weed consumption.

While the complementarity contribution of the different carabid taxa to pest predation in our results is exciting, it is important to remember that it is solely based on the data of pest DNA detection in the carabid gut. Many other factors impacting pest control remain, such as carabid abundance or metabolic rate, which bring nuances to the results. *Nebria* and *Brachinus* stood out for their higher detection rate of pests’ DNA, *Nebria* for aphids in June and *Brachinus* for aphids and slugs in April, but these genera have a really low abundance at those specific times (Figure 3c), which suggest a low impact on pest control. Bigger species are also expected to have a higher impact than smaller ones due to their higher metabolic rates, and consequently their higher food intake. A lower contribution from small species is therefore suspected from *Bembidion* and *Trechus* genera on slug predation despite their similar detection rate in May to bigger genera like *Amara*, *Poecilus* or *Harpalus*. Another limitation of our study includes the presence of potentially important sources of variation in the detection of prey DNA. It is unlikely that carabids digest all types of prey at the same rate. Previous studies on rates of digestion by *P. melanarius* have indicated that the detectability half-life is highly prey-dependent, with lower rates for aphids than for other prey (between 5–20 h in most cases [45,46]), highly variable values for earthworms that were as high as 90 h [10,47] and intermediate values between 20–40 h for slugs and spiders [11,47]. Consequently, pest consumption by carabids might therefore be underestimated when compared to alternative prey consumption. The digestion rate also varies among predator species, with Greenstone et al. [8] showing that different predator taxa had different digestion rates after eating the same quantity of Colorado potato beetles, resulting in variation in post-predation detection times. Information on the variation in digestion rates among carabids is effectively non-existent and the study of this phenomenon has largely been limited to *P. melanarius*; it is therefore difficult to estimate variations between carabid species. Variability in detection rates also depends on amplicon length or primer specificity [45]. In our multiplex PCR, primer concentrations were adjusted so that detection was similar for each prey type with an equivalent amount of DNA, mitigating biases due to amplicon length. Lastly, mistaking secondary predation events (carabid–spider–aphid) for primary predation events (carabid–aphid) could lead to an overestimation of aphid predation by carabids. While the risk is high in situations where both predators did not have the time to digest their respective prey, detection of secondary predation is greatly reduced when they do [48,49]; misinterpretation of trophic links is therefore expected to be limited in our study. An uncertainty however remains when trying to assess carabids’ predation impact due to our inability to distinguish predation events from scavenging ones with our methodology. Multiple carabid species have been shown to prefer dead prey to live ones in laboratory experiments [50,51,52], which could suggest a more saprophagous diet for carabids even in natural conditions and consequently a lower impact on prey populations. This could also explain the high detection rates of springtail DNA found in the gut of every carabid species, despite genera like *Notiophilus, Leistus* or *Loricera* having evolved specialized and crucial morphological adaptations for hunting and consuming springtails that are absent from other carabid species [53,54,55].

## 5. Conclusions

The current study contributed to a better understanding of carabids’ diet dynamics and revealed prey switching consistent with prey availability in the environment. Our results highlight the importance of alternative prey, particularly decomposers, in sustaining carabid populations in the absence of pests and in maintaining their consumption of pests over the year. Adopting agricultural practices that promote decomposers, such as mulching or conservation practices, could therefore result in a positive bottom-up effect on the natural enemy communities and consequently be beneficial to the farmers through a top-down effect on pests. However, it remains to be shown, in practice, that carabid predation results in the regulation of pest populations and therefore in a biological control service.

## Figures and Tables

**Table 1:** Analysis of deviance table testing the significance of the effect of prey group, session and their interactions on the probability of detecting prey DNA in the gut of carabids. Chisq stands for the chi-square statistic, Df for the degree of freedom and Pr (>Chisq) for *p*-values associated with the chi-square statistic.

Fixed Effect	Chisq	Df	Pr (>Chisq)
(Intercept)	75.224	1	<0.001
Prey group	302.143	4	<0.001
Session	49.939	3	<0.001
Prey group: Session	236.196	12	<0.001

## Data Availability

Data will be available on the Recherche Data Gouv Repository.

## Supplementary Materials

The following supporting information can be downloaded at: https://www.mdpi.com/article/10.3390/insects15080610/s1. Supplementary Material S1: Map of France showing the location of the five regions where carabid beetles were sampled. Supplementary Material S2: Primer pairs composing the multiplex PCR assay for assessing trophic interactions in wheat crops. Supplementary Material S3: Taxa used for testing primers’ sensitivity and specificity. Supplementary Material S4: Distribution of the number of selected carabid beetles for MGCA per taxon and session. Supplementary Material S5: Correlation plots between prey abundance and their DNA detection rate in the gut of carabid beetles for aphids, slugs and spiders, and discussion.
