# Supplementary material for: Prey Switching and Natural Pest Control Potential of Carabid Communities over the Winter Wheat Cropping Season
Source: Insects. 2024 Aug 13;15(8):610. doi: 10.3390/insects15080610 (PMC11354267; doi:10.3390/insects15080610)
Supplement: Supplementary file 1 [file insects-15-00610-s001.zip › insects-3128158-supplementary.pdf]

Supplementary Materials:

Supplementary Material S1: Map of France showing the location of the five regions where carabid beetles were sampled

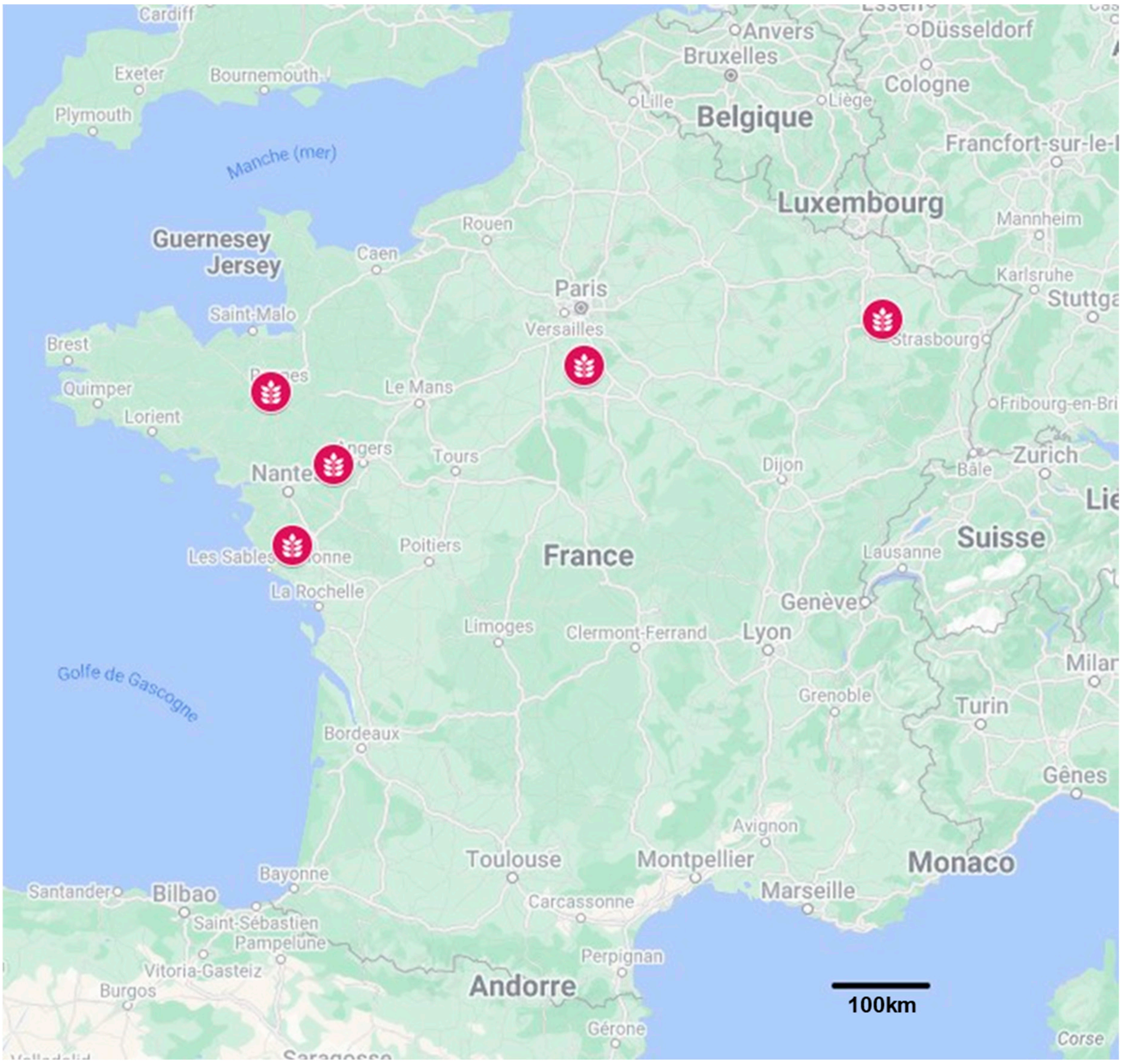

Supplementary Material S2: Primer pairs composing the multiplex PCR assay for assessing trophic interactions in wheat crops. Columns show the target group, primer name, target gene, primer sequence, expected amplicon length and final concentration of each primer in PCR

Target	Name	Gene	5′-3′ Sequence	Amplicon length (bp)	Source	Concentration (μM) in Primer mix
**Earthworms**	S408<br>A413	18S	CCATGATTTCTTAGATCGTACAATCC<br>ATARGGGTCGGAGCTTTGTG	85	Staudacher, 2016	0.2
**Springtails**	S412<br>A414	18S	CGGACGATTTTRYTRGTTCGT<br>ATGCACWAATGTTCAGGCTGTA	120	Staudacher, 2016	0.25
**Aphids**	S421<br>A424	18S	ATGTCTCAGTGCAAGCCGC<br>GCCGCGACGGGCC	205	Staudacher, 2016	0.3
**Spiders**	S407<br>A408	18S	AATAACRATACGGGACTCTTTYGAGA<br>CGAGACAACCGGTRAAGATCAT	260	Staudacher, 2016	0.2
**Slugs**	SF496<br>SR496	18S	YAGTAACGGCGAGTGAAGC<br>CGGATCCACCCGTTTACCT	360	Designed for this study	0.08

Supplementary Material S3: Taxa used for testing primers' sensitivity and specificity.

Assessment of primers' sensitivity and specificity was performed for all primer pairs during the multiplex assay. Primers' sensitivity was determined for all primer pairs in the multiplex by testing the detection of target DNA at different concentrations (250, 100, 50, 25 and 10 copies/µL of target DNA). Primers' specificity was tested by the amplification success of target (underlined) and non-target invertebrate DNA commonly found in farming systems

Class, order	Family	Species
**Acari**		
**<u>Araneae</u>**	<u>Linyphiidae</u>	<u>*Agyneta rurestris*</u>
		<u>*Oedothorax retusus*</u>
	<u>Lycosidae</u>	<u>*Pardosa agrestis*</u>
		<u>*Trochosa ruricola*</u>
	<u>Tetragnathidae</u>	<u>*Pachygnatha clercki*</u>
		<u>*Pachygnatha degeeri*</u>
**<u>Clitellata</u>**	<u>Lumbricidae</u>	<u>*Aporrectodea caliginosa*</u>
		<u>*Aporrectodea longa*</u>
		<u>*Aporrectodea tuberculate*</u>
		<u>*Lumbricus rubellus*</u>
		<u>*Octolasion lacteum*</u>
**Coleoptera**	Cantharidae	
	Carabidae	*Agonum muelleri*
		Anchomenus dorsalis
		Asaphidion flavipes
		Bembidion quadrimaculatum
		Harpalus affinis
		Metallina lampros
		Nebria brevicollis
		Poecilus cupreus
		Pterostichus melanarius
		Pterostichus niger
		Trechus secalis
	Chrysomelidae	*Phyllotreta vittula*
	Coccinellidae	*Coccinella septempunctata*
	Curculionidae	
	Elateridae	*Agriotes lineatus*
		Agriotes obscurus
		Agriotes sordidus
		Agriotes sputator

	Meloidae	
	Nitidulidae	*Meligethes aeneus.*
	Scarabaeidae	*Melolontha melolontha*
	Staphylinidae	*Tachyporus chrysomelinus*
		Xantholinus tricolor
**Collembola**		
**Diplopoda**	Julidae	
**Diptera**	Agromyzidae	*Liriomyza huidobrensis*
	Calliphoridae	
	Cecidomyiidae	
	Drosophilidae	*Drosophila suzukii*
	Syrphidae	*Episyrphus balteatus*
		Eristalis tenax
		Eristalis arbustorum
**Gastropoda**	Limacoidae	*Arion spp.*
		Deroceras spp.
**Hemiptera**	Aphididae	*Acyrthosiphon pisum*
		Rhopalosiphum padi
		Sitobion avenae
**Hymenoptera**	Formicidae	*Tetramorium spp.*
**Isopoda**		
**Lepidoptera**	Gelechiidae	*Tuta absoluta*
	Plutellidae	*Plutella xylostella*
**Lithobiomorpha**	Lithobiidae	
**Nematoda**		
**Neuroptera**	Chrysopidae	*Chrysoperla carnea*
**Orthoptera**	Gryllidae	*Gryllus campestris*
**Rodentia**	Muridae	*Mus musculus*
**Thysanoptera**	Frankliniella	

Supplementary Material S4: Distribution of the number of selected carabid beetles for MGCA per taxon and session

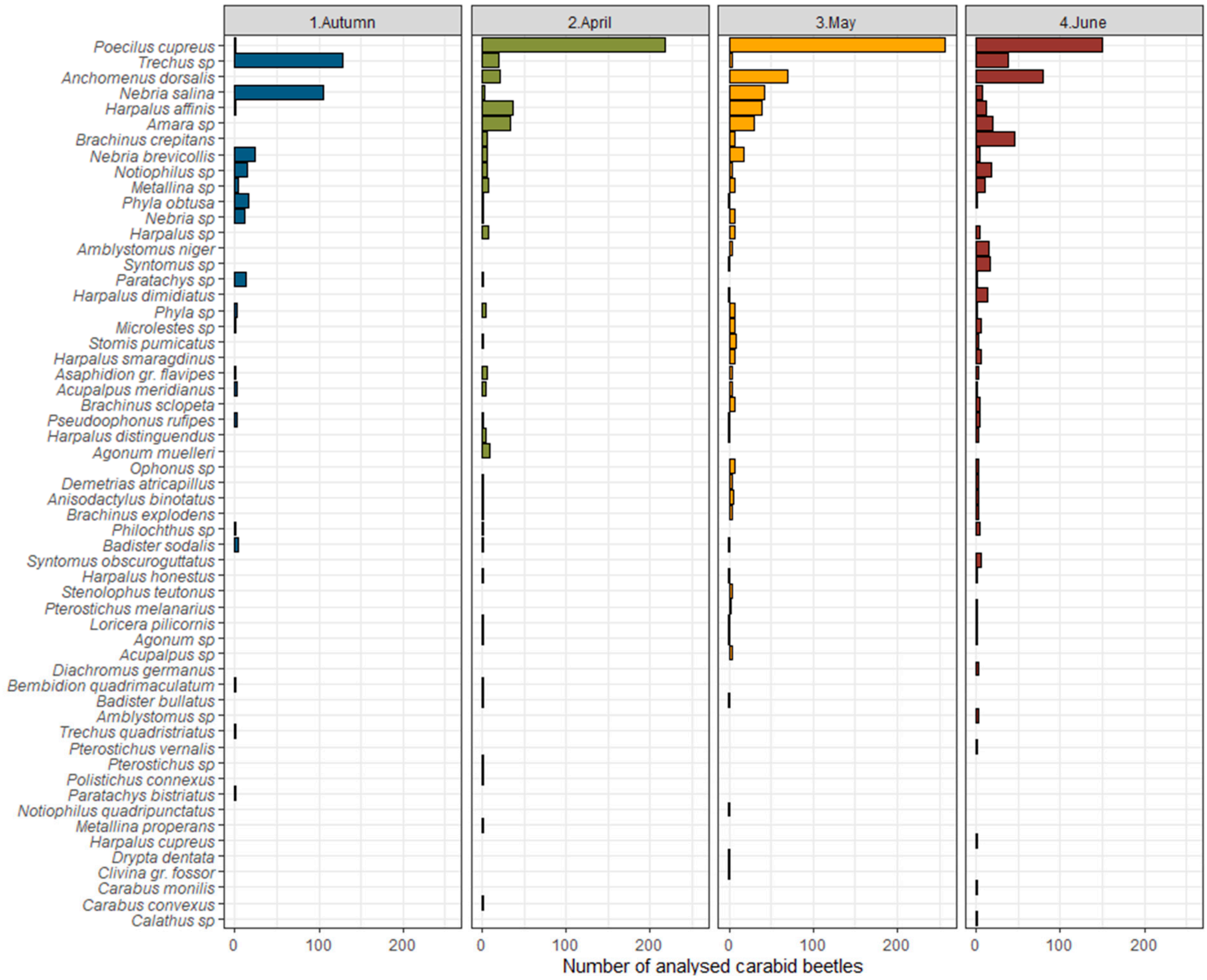

Supplementary Material S5: Correlation plots between prey abundance and their DNA detection rate in the gut of carabid beetles for aphids, slugs and spiders, and discussion.

Each dot is for a combination of session and field, referred to as point. All sessions are combined to make a single plot per group of prey but are distinguished by the dot shape. The correlation coefficient, R, is calculated with the Pearson method and the p-value, p, reflects the significance of the correlation between prey abundance and prey detection rate. The dataset for prey abundance is unfortunately incomplete, so this analysis is not included in the main text. The respective number of points for which prey abundance was measured is specified on each plot.

Methods for measuring prey abundance: 1) Aphid abundance was assessed by counting the number of aphids present on 100 randomly selected wheat tillers per point. 2) A trapping kit from De Sangosse company was used to measure slug abundance. The trap consists of a 50cm x 50cm cover that was humidified with water and left face down in the field. After one week, the number of slugs under the trap was counted. In total, four traps were used per point. 3) The abundance of spiders was estimated with pitfall traps filled with brine (100 g of salt for 1 L of water mixed with 3–4 drops of TEEPOL soap) that were open for one week. In total, four traps were used per point.

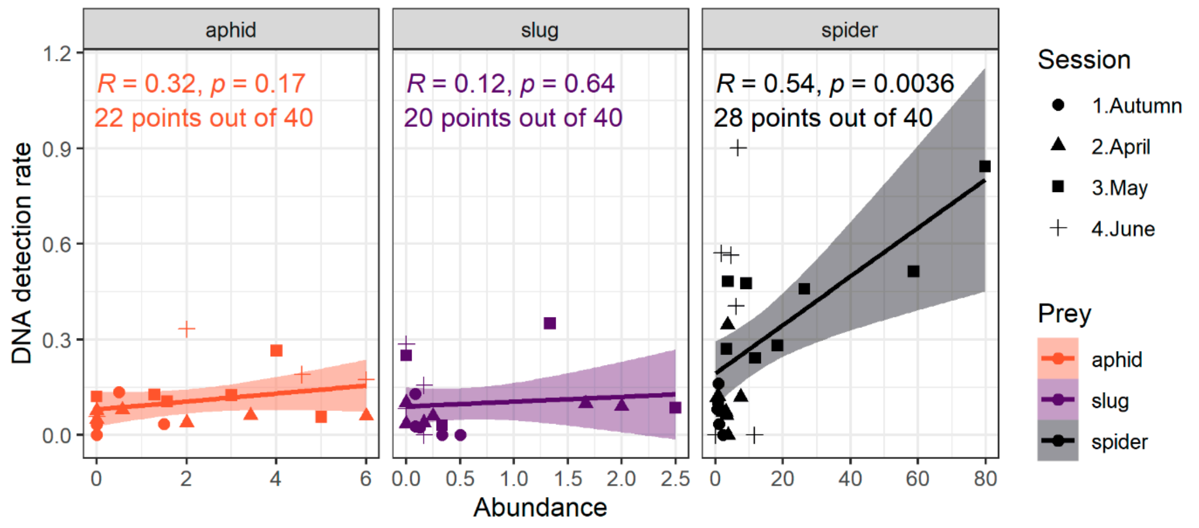

Results and discussion: It is important to note that we only have partial data on prey abundance which compromises our ability to measure the correlation. The only significant correlation is for the better-represented prey group (28 points): spider DNA detection rate is

significantly correlated to spider abundance ($P = 3.6.10^{-3}$) and its correlation coefficient is positive and moderately strong ($R = 0.54$). The correlation between pests' abundance and their detection rate in carabids' gut was not significant and their correlation coefficient was weak for aphids ($R = 0.32$) to very weak for slugs ($R = 0.12$). Multiple reasons are supposed to be at play here: the first one is the low number of points for pests, in barely half the combination of session-field was the pests abundance measured. We suspect that it was particularly detrimental to determining the aphid correlation. The low pest infestation of the 2018-2019 crop season in France is another factor that comes to mind since it reduces the potential distribution of our data. The last two reasons concern mainly the prey group of slugs and is a matter of methodology. First, slug trapping was very dependent on the weather and hydrometry of the time the trap was collected, which probably biased our estimation of slug abundance between different points. Second, one of our suspicions that is indicated in the main text is that carabid beetles actually consume slug eggs (or juveniles) for which we have no information.
